# Suicidal behaviour among youths associated with psychopathology in both parents and youths attending outpatient psychiatric clinic in Kenya

**DOI:** 10.1186/1744-859X-12-13

**Published:** 2013-04-27

**Authors:** Lincoln I Khasakhala, David M Ndetei, Muthoni Mathai

**Affiliations:** 1 Department of Psychiatry, University of Nairobi, P.O. Box 30197, Nairobi, Kenya; 2 Africa Mental Health Foundation (AMHF), P.O. Box 59176 00200, Nairobi, Kenya

**Keywords:** Suicidal behaviour, Psychiatric disorders, Youth, Parents, Outpatient, Kenya

## Abstract

**Background:**

Suicide is a major cause of death among youths particularly with psychiatric, alcohol abuse and substance abuse disorders. There are relatively few studies on the relationship between psychiatric and substance abuse disorders with suicidal behaviour from low-income countries. This study examines the relationship between suicidal behaviour and co-existing psychiatric or substance disorders among youths and depressive and alcohol use disorders in their parents.

**Method:**

The study sample had 678 respondents: 250 youths and 226 and 202 biological mothers and fathers, respectively.

**Results:**

This study found a significant statistical association between depressive (*p* < 0.001), alcohol abuse (*p* <0.001) and substance abuse (*p* < 0.001) disorders and suicidal behaviour in youths. There was a significant relationship between maternal depressive disorder (*p* < 0.001) and perceived maternal rejecting parenting behaviour (*p* < 0.001) with suicidal behaviour in youths. There was a greater odds of a youth with two to three (odds ratio (OR) = 3.63; *p* = 0.009) and four or more (OR = 8.23; *p* < 0.001) co-existing psychiatric disorders to have suicidal behaviour than a youth with only one psychiatric disorder. The results also indicate that a higher proportion of youths between ages 16–18 years had suicidal behaviour than youths below 16 years or above 18 years of age (*p* = 0.004).

**Conclusion:**

These findings suggest that youths with psychiatric and substance abuse disorders have mothers living with a depressive disorder. Also, perceived maternal rejecting parenting behaviour contributes significantly to the development of suicidal behaviour later in adolescent years.

## Introduction

Worldwide, suicide is among the top five causes of mortality in the 15- to 19-year-olds [1,2]. It is the third leading cause of death worldwide in people aged 15–34 years, re-identified to have 1.4% of disease burden globally [3,4]. Previous studies show that youths with a history of admission or attendance to a medical facility come from a dysfunctional family or have parents with a psychiatric illness [5-7]. These studies on the history of admission or attendance to a medical facility also show that the affected youths aged 10–19 years have an increased risk of developing suicidal behaviour.

The relationship between psychiatric disorders or substance abuse and suicidal behaviour among youths has been studied in the developed world, with few studies from low- and middle-income countries [8,9]. Research findings indicate that depressive disorder is associated with suicidal behaviour [10]. Unfortunately, if depression remains untreated, co-morbid disorders: anxiety, bipolar mood and substance use disorders, develop which are associated with severe suicidal behaviour [11-13]. Studies have further documented that youths with substance abuse disorders have higher rates of depression (15%–24%) than youths in the general population (2%–8%) [14-16]. It has also been shown that youths diagnosed with co-morbid disorders have severe substance abuse with poorer drug treatment outcomes [16]. These youths with psychopathology come from dysfunctional families where one or both parents have psychiatric or substance use problems associated with maladaptive parenting behaviour [17-22]. Bridge et al. showed that between 80% and 90% of adolescents who had suicide plan and/or had attempted suicide had co-morbid psychiatric disorders [23]. In 2004, Wilcox et al. showed that there is an association between completed suicide and drug abuse [24]. Therefore, the co-morbidity of psychiatric disorders, particularly of mood, disruptive, and substance abuse disorders, significantly increases the risk for youths to develop suicidal behaviour.

In Africa, Ndosi identified schizophrenia, substance abuse, HIV/AIDS and personality disorders as co-morbid psychiatric and medical conditions associated with suicidal behaviour [25]. Ndosi indicated that 90% of people who commit suicide have co-morbid psychiatric disorders, although social factors have a predominant influence on their suicidal behaviour [25]. Existing data from the South African Stress and Health Study on the prevalence and correlates of suicidal behaviour revealed that having a psychiatric disorder is a risk factor for suicidal behaviour [26]. In this South African study [26], respondents with at least one Diagnostic and Statistical Manual of Mental Disorders - Fourth Edition (DSM-IV) disorder were four times more likely to attempt suicide, while those with three or more disorders were eight times more likely to attempt suicide or develop suicidal ideation than those with no psychiatric disorder. This paper is written in order to document the association between psychiatric disorders in youths and their parents and perceived parenting behaviour and suicidal behaviour in Kenya.

## Methods

### Respondents

The study respondents were recruited from the Youth Centre of Kenyatta National Hospital (KNH), which is a teaching and referral centre in Kenya and admits youths aged 13–25 years. Inclusion criteria consisted of first identifying a youth with any axis 1 psychiatric disorder or substance use disorder registered at the outpatient clinic through psychological interview and explaining the purpose of the study. The next appointment was scheduled where both parents of the youth were to be interviewed at the clinic. The youths informed their parents about the purpose of the study and were accompanied by their parents on the second day of the interview to the outpatient clinic. The parents and their youths were first provided with a description of the study before signing an informed consent, but youths who were below 18 years of age signed an ascent form while one parent signed their informed consent. Six hundred and seventy-six respondents who included 250 youths and 226 and 202 biological mothers and fathers, respectively, were recruited in the study between September 2007 and May 2009. Approval for data collection was obtained from KNH and the University of Nairobi Ethics and Review Board.

### Diagnostic and perceived parenting behaviour assessment

Respondents’ psychiatric disorders were assessed using psychiatric structured interview protocols: the Mini International Neuropsychiatric Interview for Children and Adolescents (MINI Kid) and Mini International Neuropsychiatric Interview for Adults (MINI Plus) which were administered to the youths and parents, respectively [27,28]. Both structured diagnostic interview schedules were developed for the diagnosis of DSM-IV psychiatric disorders [29]. These structured questionnaires are designed to meet the need for a short but accurate structured psychiatric interview for multi-centre clinical trials and epidemiologic studies and have been tested and shown to have high validity and reliability in diagnosing a DSM-IV-TR axis I disorder [27-29]. These schedules were used as a first step in outcome tracking and confirming axis 1 DSM-IV disorders and the severity of suicidal behaviour among the respondents.

Perceived parenting behaviour by youths was assessed using the *Egna Minnen Betraffande Uppfostran* (EMBU) questionnaire (English translation from Swedish is ‘own memories of childhood upbringing’) [30,31]. This is a self-administered questionnaire about perceived parenting behaviour where respondents recall in what way their parents were alike and in what way the parents differed using the questionnaire that has 81 items. The 81 items in the questionnaire measure two constructs that has a total of eight factors: four parenting styles and forms of child abuse which are further computed into four types of parenting behaviours [30,31]. The 81 items were answered separately for the mother and father according to Likert-type categories: ‘no, never’ = 1, ‘sometimes’ = 2, ‘yes, often’ = 3 and ‘yes, always’ = 4 [31]. From each of these four scales, the eight items are selected for showing the highest factor loading: ‘rejection or punishment’, ‘emotional warmth or rejection’ and ‘control or overprotection’. This was adopted from a factor analysis and trans-culturally generalizable factor structure, with the scales ‘rejection’, ‘emotional warmth’ and under-protection [31]. The questions are grouped into eight constructs following factor analysis: parental emotional abuse/neglect, parental physical abuse/neglect, authoritarian parent, under-involved parent, permissive parent and authoritative parent. The constructs were further factually derived into four perceived parenting behaviour dimensions: emotionally attached parent (emotionally/physically abusing and authoritarian parenting behaviour), rejecting parent (emotionally and physically neglecting parenting behaviour), under-protective parent (permissive or under-involved parenting behaviour) and adaptive parenting behaviour (authoritative parenting behaviour), which are distributed across the four scales [30,31]. This questionnaire is self-administered and has been used in Kenya in a similar age group population [32].

The socio-demographic questionnaire was filled out in the presence of both youths and parent(s). The structured psychiatric interview schedule and EMBU for each participant were conducted confidentially one-on-one but later were matched.

### Statistical analysis

Data of the MINI Kid and MINI Plus were computed selectively for cases that illustrated themes that classify the following: birth position and how long the youth has had the psychiatric disorder and each DSM-IV disorder category [29]. This was done by selecting all ‘yes’ responses for each respondent on the structured interview schedule for each specific psychiatric disorder. A specific axis 1 DSM-IV disorder for each respondent was arrived at if the ‘yes’ responses met the criterion for the specific psychiatric disorder. During selective coding to make a DSM-IV axis I disorder [29], common (depressive, anxiety and substance/alcohol abuse disorders) and severe (schizophrenia and bipolar 1 mood disorders) disorders ultimately guided the analysis. Thereafter, the data were reorganized into specific DSM-IV categories to build on major quantifiable DSM-IV axis I psychiatric disorders [29]. The proportions of youth and parent respondents were broken down according to DSM-IV diagnostic criteria to compare the clinical classification of the psychiatric disorders delineated and analyzed their associations with perceived parenting behaviour. Bivariate and multivariate statistical analyses were done to test the association between perceived parenting behaviour and psychopathology among the respondents including suicidal behaviour controlling for gender and age of youths.

## Results

A total number of 267 youths were recruited, but only 250 completed the assessment: psychological interview, MINI Kid structured interview and self-administered EMBU questionnaire. Out of those who did not complete (4.5%) [11], five did not return to the clinic for follow-up despite several telephone reminders about their appointment, four parents did not sign up the consent forms and three youths had severe psychotic disorder and therefore did not fill out the EMBU questionnaire. The other five youths were not included in the analyses: two had post-traumatic stress disorder (PTSD) as a result of sexual abuse within their family setting and did not want their parent involved in the study, and three others had witnessed their family members being murdered and therefore had traumatic grief and could not complete the interviews. Out of the 250 total number of mothers expected to participate, 90.4% (226) were recruited: 9 fathers were widowers, 2 mothers were unknown to their children and 12 lived upcountry in their rural homes and therefore were unavailable. Among a total of 250 fathers expected to be recruited in the study, 80.8% (202) were recruited: 38 mothers were widows while four fathers were unknown to their children. The age range of the youth who completed the study was 13 years for the youngest and 22 years for the oldest with a mean age of 16.92 years, median of 17 years and standard deviation of 2.151 (Table 1).

**Table 1:** DSM-IV axis disorders identified from respondents

**DSM-IV axis 1 disorder**	**Maternal (*N *= 250)**		**Paternal (*N *= 250)**		**Youth (*N *= 250)**	
	***n***	**%**	***n***	**%**	***n***	**%**
Major depressive disorder						
Identified	117	47.8	38	15.5	133	53.2
Absent	111	45.3	160	65.3	117	46.8
Not assessed^a^	17	6.9	47	19.2	–	–
PTSD						
Identified	5	2.0	2	0.8	22	8.8
Absent	223	91.0	196	80.0	238	91.2
Not assessed^a^	17	6.9	47	19.2	–	–
Alcohol use						
Identified	3	1.2	96	39.2	117	46.8
Absent	225	91.8	102	41.6	133	53.2
Not assessed^a^	17	6.9	47	19.2	–	–
Schizophrenia						
Identified	–	–	–	–	15	6.0
Not identified					235	94.0
Bipolar mood disorder						
Identified	–	–	–	–	23	9.2
Not identified	–	–	–	–	227	90.8
Multiple drug abuse						
Identified	–	–	–	–	18	9.0
Not identified	–	–	–	–	232	91.0
Conduct disorder						
Identified	–	–	–	–	21	8.4
Not identified	–	–	–	–	229	91.6
Suicidal behaviour						
Identified	–	–	–	–	205	82.0
Not identified	–	–	–	–	45	18.0
Any other anxiety disorder						
Identified	–	–	–	–	45	18.0
Not identified	–	–	–	–	205	82.0
Dysthymia						
Identified	43	17.6	–	–	–	–
Absent	185	75.5	–	–	–	–
Not assessed^a^	17	6.9	–	–		

^a^Parent either not recruited or deceased.

Table 2 indicates the results of suicidal behaviour among youths in relation to background characteristics, whereby 82% of the youth had severe suicidal behaviour (plans and attempts). The age 16–18 years was significantly associated with severe suicidal behaviour (OR = 3.31; 95% CI, 1.47–7.47; *p* = 0.004) compared to age 19–22 years.

**Table 2:** Suicidal behaviour among youths in relation to their background characteristics

**Variables**	**Suicidal behaviour**		**No suicidal behaviour**		**OR**	**95% CI**		*p *value*
	**(*N *= 205)**		**(*N *= 40)**					
	***n***	**%**	***n***	**%**		**Lower**	**Upper**	
Age in years								
13–15	50	82.0	11	18.0	1.73	0.73	4.13	0.216
16–18	113	89.7	13	10.3	3.31	1.47	7.47	*0.004*
19–22	42	72.4	16	27.6	Reference			
Sex								
Female	82	82.0	18	18.0	0.81	0.41	1.61	0.556
Male	123	84.8	22	15.2	Reference			
Position of birth								
Only child/first born	94	84.7	17	15.3	2.21	0.75	6.50	0.149
Second born	49	89.1	6	10.9	3.27	0.92	11.64	0.068
Third born	28	77.8	8	22.2	1.40	0.41	4.79	0.592
Fourth born	19	86.4	3	13.6	2.53	0.54	11.85	0.238
Fifth born or higher	15	71.4	6	28.6	Reference			
Level of education								
Secondary	132	88.0	18	12.0	2.08	0.97	4.42	0.05
College	20	74.1	7	25.9	0.81	0.29	2.27	0.687
Primary	53	77.9	15	22.1	Reference			
Marital status of parents								
Single mother, never married	13	81.3	3	18.8	0.88	0.24	3.27	0.847
Widower/widow	33	82.5	7	17.5	0.96	0.39	2.36	0.922
Married	148	83.1	30	16.9	Reference			
Duration of illness								
1–12 months	60	81.1	14	18.9	0.67	0.22	2.03	0.478
>1–5 years	107	83.6	21	16.4	0.80	0.28	2.28	0.671
>5 years	32	86.5	5	13.5	Reference			

*Significance at *p* < 0.05 (italicized); OR, odds ratio; 95% CI, 95% confidence interval.

Table 3 indicates the results of suicidal behaviour among youths assessed using the MINI Kid questionnaire [27,28] in relation to perceived parenting behaviour assessed using the EMBU questionnaire [30,31] and DSM-IV axis I disorders among parents using the MINI Plus interview schedule [30,31]. Six factors were identified to associate significantly with the occurrence of suicidal behaviour among youths: rejecting behaviour in mothers (OR = 3.52; 95% CI, 1.17–10.64; *p = 0.026*), major depressive disorder (MDD) in mothers (OR = 2.14; 95% CI, 1.05–4.37; *p = 0.037*), presence of any psychiatric disorder in mothers (OR = 2.29; 95% CI, 1.11–4.72; *p = 0.024*), death of fathers with reference to non-use of alcohol in fathers (OR = 3.13; 95% CI, 1.02–9.64; *p = 0.047*), death of fathers with reference to the absence of any disorders among fathers (OR = 4.75; 95% CI, 1.49–15.12; *p = 0.008*) and presence of any disorder in fathers (OR = 3.09; 95% CI, 1.47–6.49; *p = 0.003*).

**Table 3:** Suicidal behaviour among youths in relation to their parents’ characteristics

**Parental variables**	**Suicidal behaviour**		**No suicidal behaviour**		**OR**	**95% CI**		*p *value*
	**(*N *= 205)**		**(*N *= 40)**					
	***n***	**%**	***n***	**%**		**Lower**	**Upper**	
Perceived maternal parenting behaviour								
No emotional connectedness	22	78.6	6	21.4	1.57	0.42	5.85	0.501
Rejecting	115	89.1	14	10.9	3.52	1.17	10.64	*0.026*
Under-protective	42	77.8	12	22.2	1.50	0.47	4.74	0.490
Normal	14	70.0	6	30.0	Reference			
Mothers: major depressive disorder								
Major depressive disorder	103	88.0	14	12.0	2.14	1.05	4.37	*0.037*
Not assessed	16	94.1	1	5.9	4.65	0.59	36.81	0.145
No major depressive disorder	86	77.5	25	22.5	Reference			
Mothers: dysthymia								
Dysthymia	36	83.7	7	16.3	1.08	0.44	2.63	0.873
Not assessed	16	94.1	1	5.9	3.35	0.43	26.15	0.250
No dysthymia	153	82.7	32	17.3	Reference			
Perceived paternal parenting behaviour								
No emotional connectedness	89	85.6	15	14.4	1.35	0.44	4.11	0.599
Rejecting	42	85.7	7	14.3	1.36	0.39	4.80	0.629
Under-protective	10	71.4	4	28.6	0.57	0.13	2.58	0.464
Normal	22	81.5	5	18.5	Reference			
Unknown	42		9					
Fathers: major depressive disorder								
MDD	35	92.1	3	7.9	3.03	0.88	10.47	0.080
Not assessed	43	91.5	4	8.5	2.79	0.94	8.34	0.066
No MDD	127	79.4	33	20.6	Reference			
Fathers: alcohol use disorder								
Alcohol use	83	86.5	13	13.5	1.86	0.88	3.92	0.104
Not assessed	43	91.5	4	8.5	3.13	1.02	9.64	*0.047*
No alcohol use	79	77.5	23	22.5	Reference			

*Significance at *p* < 0.05 (italicized); OR, odds ratio; 95% CI, 95% confidence interval.

Suicidal behaviour among youths was significantly associated with MDD (OR = 5.27; 95% CI, 2.39–11.66; *p < 0.001*), any drug abuse (OR = 6.66; 95% CI, 2.81–15.75; *p < 0.001*) and alcohol use (OR = 6.69; 95% CI, 2.69–16.63; *p < 0.001*), as tabulated in Table 4.

**Table 4:** Suicidal behaviour among youths in relation to their other mental health status

**Variables**	**Suicidal behaviour**		**No suicidal behaviour**		**OR**	**95% CI**		*p *value*
	**(*N *= 205)**		**(*N *= 40)**					
	***n***	**%**	***n***	**%**		**Lower**	**Upper**	
Major depressive disorder								
Yes	124	93.2	9	6.8	5.27	2.39	11.66	*<0.001*
No	81	72.3	31	27.7	Reference			
Conduct disorder								
Yes	20	95.2	1	4.8	4.22	0.55	32.36	0.134
No	185	82.6	39	17.4	Reference			
Any anxiety disorder								
Yes	32	71.1	13	28.9	0.38	0.18	0.82	*0.012*
No	173	86.5	27	13.5	Reference			
Any drug abuse								
Yes	120	94.5	7	5.5	6.66	2.81	15.75	*<0.001*
No	85	72.0	33	28.0	Reference			
Alcohol use								
Yes	111	94.9	6	5.1	6.69	2.69	16.63	*<0.001*
No	94	73.4	34	26.6	Reference			

*Significance at *p* < 0.05 (italicized); OR, odds ratio; 95% CI, 95% confidence interval.

Suicidal behaviour among youths was significantly associated with two to three co-morbid disorders (OR = 3.63; 95% CI, 1.37–9.62; *p = 0.009*) and four or more co-morbid disorders (OR = 8.23; 95% CI, 3.37–20.13; *p < 0.001*), as shown in Table 5.

**Table 5:** Youth suicidal behaviour in relation to their patterns of psychiatric condition

**Variables**	**Suicidal behaviour**		**No suicidal behaviour**		**OR**	**95% CI**		*p *value*
	**(*N *= 205)**		**(*N *= 40)**					
	***n***	**%**	***n***	**%**		**Lower**	**Upper**	
1 disorder	52	65.8	27	34.2	Reference			
2–3 disorders	42	87.5	6	12.5	3.63	1.37	9.62	*0.009*
4 or more disorders	111	94.1	7	5.9	8.23	3.37	20.13	*<0.001*

*Significance at *p* < 0.05 (italicized); OR, odds ratio; 95% CI, 95% confidence interval.

As tabulated in Table 6, predictors of suicidal behaviour among youths include MDD (AOR = 4.63; 95% CI, 1.68–12.73; *p = 0.003*), alcohol use (AOR = 4.25; 95% CI, 1.49–12.14; *p = 0.007*) and any drug abuse (AOR = 5.23; 95% CI, 1.88–13.91; *p < 0.001*). Any anxiety disorder was associated with reduced cases of suicidal behaviour (AOR = 0.20; 95% CI, 0.07–0.59; *p = 0.003*).

**Table 6:** Predictors of suicidal behaviour among youths

**Predictors**	**AOR**	**95% CI**		*p *value*
		**Lower**	**Upper**	
Major depressive disorder				
Yes	4.63	1.68	12.73	*0.003*
No	Reference			
Any anxiety disorder				
Yes	0.20	0.07	0.59	*0.003*
No	Reference			
Alcohol use				
Yes	4.25	1.49	12.14	*0.007*
No	Reference			
Any drug abuse				
Yes	5.23	1.88	13.91	*<0.001*
No	Reference			

*Significance at *p* < 0.05 (italicized); AOR, adjusted odds ratio; 95% CI, 95% confidence interval.

## Discussion

Suicidal behaviour is considered a worldwide problem and an increasing source of concern. This study shows a significant tendency toward an increased rate of severe suicidal behaviour (prevalence of 82%) among youths with psychiatric disorders which has been documented in previous studies [10,23,24].

The family dysfunctions in this study include the following: youths from a single-parent family (never married, separated widows/widowers), youths who have parents with psychiatric disorders (alcohol use, mood disorders), a family history of severe suicidal behaviours and violent and abusive family structures (parenting behaviour with no emotional attachment), maladaptive maternal rejecting parenting behaviour, parents' inadequate authority (under-protective parenting behaviour) and parents’ lack of time to observe and deal with the child’s emotional distress. These findings are comparable to previous studies that have shown that mental health problems compromise a mother’s or a father’s parenting abilities, and this poses a threat to their youths’ adjustment and behaviour [17,18]. Thus, a parent with a psychiatric disorder has also a maladaptive parenting behaviour. The perceived maternal-parental rejection or paternal under-protection obstructs interaction between parents and their children. The parenting behaviour in such a family setting is perceived by children to be a poor emotional expression: ‘I have no interest in you’. This, therefore, disconnects children from their parents, creating a barrier for children to explore and form connecting bonds with their parent(s). This un-connectedness between the child and a parent leads to confusion, conflict and frustration in the growing child, a precursor for a youth/child to develop psychopathology and suicidal behaviour.

These findings are comparable to evidence from previous studies which have documented that parents of depressed youths also have depression [33-35]. These relationships seen in other studies are similar in this outpatient setting in Kenya. These findings are also consistent with other studies which assessed the co-morbidity of psychiatric disorders and showed a significantly increased risk for youth suicidal behaviour [5,6,10,23,36]. It is also comparable to a study by Beautrais et al., which examined precipitating factors and life events in serious suicide attempts among youths aged 13 through 24 years [37]. Also, these relationships between maternal rejecting behaviour and depression and offspring suicidality may suggest the need for therapeutic approaches that treat the family unit, not just individuals separately, and the relationship between paternal death and child suicidal behaviour may suggest that those whose parents die should receive targeted suicide prevention programming.

Another important finding of this study is the relationships between psychopathology (MDD and alcohol/substance use disorder) and suicidal behaviour among youths that did not show any significant difference according to gender. This striking finding emerging from this study is the extent to which suicidal behaviour co-exists with psychiatric disorders among youths. Majority of the youths had severe psychiatric disorders (depression, alcohol use problems, multiple substance use disorders and anxiety disorders). Unlike in earlier studies, women were more likely to engage in suicidal behaviours than men, probably because they had a higher prevalence of depression, which was a strong predictor of suicide attempts. In this study, alcohol dependence in youths may be the equivalent of depression or anxiety. These are externalizing syndromes which may have been un-documented in previous studies and therefore may explain why there is no difference in gender in this study. This was documented by Buglass and Horton in 1974 and Appleby in 1992 [38,39].

It can be postulated, therefore, that youths resort to suicidal behaviour so that they can externalize their problems, which is a reflection of increased risk associated with the co-morbidity of internalizing axis disorders, such as depression and anxiety disorders.

The results also indicate that the presence of multiple disorders is associated with an increased risk for suicidal behaviour (two to three co-morbid disorders and four or more co-morbid disorders compared to only one disorder). This indicates that the increasing presence of co-morbid psychiatric disorders increases the number of psychiatric symptoms and therefore difficulty to bear, hence the increased odds of suicidal behaviour. This is consistent with previous study findings [32,39-41] which have documented that suicidal behaviour co-exists among patients with multiple psychiatric disorders. This finding is also similar to several surveys which have indicated that up to three-quarter of those who eventually take their own lives show one or more symptoms of depression co-existing with other psychiatric disorders or substance abuse disorders [15,16]. These results are comparable to other findings which have revealed that youths who use alcohol and illicit drugs (multiple substance use) have co-morbid depressive disorder and are identified among adolescents who commit suicide [15,16,42,43]. Nonetheless, these results suggest that gender differences in the link between psychopathology and suicide should be explored further in other samples in which rates of suicide attempts are higher, especially among male respondents.

Certain limitations of the current study should be considered in interpreting the findings. The diagnosis and symptom assessment relied on the DSM-IV criteria. Future research should confirm these results using the most recent version of DSM. However, the use of the DSM-IV criteria allowed comparative data to be obtained from both parents and youths who were assessed concurrently for psychopathological symptoms, increasing the reliability of our statistics. An additional weakness of the current study is the binary index (yes/no) of both psychiatric disorders and suicidal behaviour that was utilized as sum criteria in making the DSM-IV diagnoses. Another weakness of this study is that we could not fully examine the important relationship between suicide and symptoms of anxiety disorders, conduct disorder, schizophrenia and bipolar disorder because of the low base rates of these syndromes in the studied population.

## Conclusion

These results have clinical implications. In general, this work informs the development of interventions to prevent individuals from engaging in behaviours that are destructive to the self and to others. Psychiatric and substance abuse disorders are strong predictors of suicidal behaviour, and these associations are more often pronounced when there is more than one co-existing psychiatric or substance abuse disorders. This suggests some universality of the relevant mechanisms underlying the genesis of suicidal behaviour. Suicidal behaviour is therefore a common problem among youths identified with psychiatric or substance abuse disorder, and these results suggest that clinicians and health care providers would improve care for their patients’ benefit from paying closer attention to the assessment of suicidal impulses in youths seen with psychiatric disorders. The findings in this study can therefore be used to develop a more comprehensive understanding and assessment of adolescent suicide risk factors, which may promote early assessments that lead to targeted interventions, thus reducing future risk of poor psychiatric health outcomes and suicidal behaviours.

## Competing interests

This work is based on a PhD study by the first author.

## Authors’ contributions

LIK was responsible for the conception, design and analysis, interpretation of data and drafting of the content. DMN was the first supervisor of the PhD study and responsible for the approval of the version to be published. MM was the second supervisor of the PhD study and responsible for the revision of the intellectual contents. All authors read and approved the final manuscript.
